# Carriage of methicillin-resistant *Staphylococcus aureus* in children <6 years old: a retrospective follow-up study of the natural course and effectiveness of decolonization treatment

**DOI:** 10.1093/jac/dkae036

**Published:** 2024-02-09

**Authors:** Thomas Helbo, Jonas Bredtoft Boel, Mette Damkjær Bartels, Magnus Glindvad Ahlström, Barbara Juliane Holzknecht, Helle Brander Eriksen

**Affiliations:** Department of Clinical Microbiology, Copenhagen University Hospital—Herlev and Gentofte, Herlev, Denmark; Department of Clinical Microbiology, Copenhagen University Hospital—Herlev and Gentofte, Herlev, Denmark; Copenhagen University Hospital—The Hospital Pharmacy, Copenhagen, Denmark; Department of Clinical Microbiology, Copenhagen University Hospital—Amager and Hvidovre, Hvidovre, Denmark; Department of Clinical Medicine, University of Copenhagen, Copenhagen, Denmark; Department of Clinical Microbiology, Copenhagen University Hospital—Herlev and Gentofte, Herlev, Denmark; Department of Clinical Microbiology, Copenhagen University Hospital—Herlev and Gentofte, Herlev, Denmark; Department of Clinical Medicine, University of Copenhagen, Copenhagen, Denmark; Department of Clinical Microbiology, Copenhagen University Hospital—Herlev and Gentofte, Herlev, Denmark

## Abstract

**Background:**

Decolonization treatment of MRSA carriers is recommended in Denmark, except in households with MRSA-positive children <2 years old (wait-and-see approach).

**Objectives:**

To investigate a wait-and-see approach in children 2–5 years old, and the effect of decolonization treatment of MRSA carriage in all children <6 years old.

**Patients and methods:**

In this retrospective follow-up study, we included MRSA carriers <6 years old in the Capital Region of Denmark from 2007 to 2021. Data were collected from laboratory information systems and electronic patient records. We divided children into age groups of <2 years or 2–5 years and decolonization treatment versus no treatment. Treatment was chlorhexidine body washes and nasal mupirocin, sometimes supplemented with systemic antibiotics. Children were followed until becoming MRSA free, or censoring. The probability of becoming MRSA free was investigated with Cox regression (higher HRs indicate faster decolonization).

**Results:**

Of 348 included children, 226 were <2 years old [56/226 (25%) received treatment] and 122 were 2–5 years old [90/122 (74%) received treatment]. Multivariable analyses did not show a larger effect of decolonization treatment versus no treatment in <2-year-olds (HR 0.92, 95% CI 0.52–1.65) or 2–5-year-olds (HR 0.54, 95% CI 0.26–1.12). Without treatment, 2–5-year-olds tended to clear MRSA faster than <2-year-olds (HR 1.81, 95% CI 0.98–3.37).

**Conclusions:**

We did not find a larger effect of decolonization treatment versus no treatment in children <6 years old, and 2–5-year-olds tended to become MRSA free faster than <2-year-olds. These results support a wait-and-see approach for all children <6 years old, but further studies are needed.

## Introduction

MRSA can be asymptomatically carried or the cause of skin and soft tissue infections and severe infections, which can be difficult to treat with antibiotics.^1^

Some of the factors associated with prolonged MRSA carriage and decolonization treatment failure are throat carriage, skin lesions, many colonized anatomical sites and positive household members.^2–5^ Knowledge about the effectiveness of MRSA decolonization treatment in children is sparse.

The prevalence of MRSA in Northern Europe overall is low.^6^ In Denmark, the incidence increased from 661 cases in 2007 to around 3500 cases per year in the pre-COVID-19-pandemic years, including approximately 350 cases per year in children ≤4 years old.^7^ In 2021, 44% of new cases were community acquired, 35% livestock associated, 11% hospital acquired or healthcare associated, and 9% imported according to data based on notifications.^8^ Twenty-seven MRSA outbreaks in Danish neonatal ICUs (NICUs) have been reported between 2008 and 2019, comprising a total of 554 individuals.^9^

Since 2006, MRSA cases have been notifiable,^10^ and the management of MRSA has been regulated by a national guideline issued by the Danish Health Authority. Decolonization treatment of carriers and household contacts is recommended to maintain a low prevalence (search-and-destroy strategy). In 2012, recommendations were changed to a wait-and-see approach in households with MRSA-positive children <2 years old, since infections related to colonization were rare and the effect of decolonization treatment in neonates seemed to be low.^11^ This change enabled us to examine children <2 and 2–5 years old, who were, per standard practice, given or not given decolonization treatment.

In this retrospective follow-up study, using time-to-event analyses, we aimed to compare the probability of becoming MRSA free with and without decolonization treatment in children <2 years old and 2–5 years old, with the purpose to investigate if the wait-and-see approach could also be rational for children 2–5 years old.

## Materials and methods

### Setting

This study was conducted in the Capital Region of Denmark, an urban region with about 1.9 million inhabitants (2021).^12^ The study included data from January 2007 until August 2021. Three departments of clinical microbiology analyse microbiological samples from the 11 hospitals in the region. Two departments also analyse all samples from primary care and advise both hospitals and GPs regarding MRSA-related infections and decolonization treatment. This study drew data from all three departments of clinical microbiology in the region.

### Decolonization regimens

Standard decolonization treatment consists of 5 days with mupirocin 2% nasal ointment applied thrice daily and a daily 4% chlorhexidine soap bodywash. Treatment includes cleaning and washing of towels and bed linen.^11^ Typically, the GP initiates the first two rounds of treatment and only refers the family to the Department of Clinical Microbiology in case of treatment failure. Standard treatment may be prolonged to 10 days and can be supplemented by systemic antibiotics, typically clindamycin if susceptible. Treatment is provided free of charge. Screening is recommended 1 and 6 months after decolonization treatment. It is standard practice to declare a person MRSA free if there has not been MRSA in the person’s samples for >6 months. Since 2012, for households with colonized children <2 years old, decolonization treatment has not been offered as standard, and screening is performed 1–2 times yearly until the child turns 2 years old.^11^

### Study design, and inclusion and exclusion criteria

This study was a retrospective follow-up study. Children were eligible for inclusion if they met all the following criteria: (i) age <6 years at the time of the first positive MRSA sample; (ii) first positive MRSA sample between 1 January 2007 and 27 February 2021; and (iii) at least one positive MRSA sample analysed at the Department of Clinical Microbiology, Herlev and Gentofte Hospital, Copenhagen University Hospital, Denmark.

Children were excluded from the study if: (i) the first positive sample was a clinical sample and there were no subsequent positive screening samples within the following 180 days (i.e. not MRSA carriers); and (ii) children with a follow-up time of <180 days after the first positive sample or the first decolonization treatment.

### MRSA screening procedures

To detect MRSA carriage, samples are routinely collected from the nose, throat and optionally perineum. Samples from wounds, chronic skin conditions and foreign bodies, such as tubes, drains and catheters are also recommended when relevant.

### Laboratory methods

Specimens were incubated overnight in an in-house-produced selective enrichment broth (tryptic soy broth with 2.5% NaCl, 3.5 mg/L cefoxitin and 20 mg/L aztreonam) and subsequently cultured on a selective chromogenic agar (CHROMagar^TM^ MRSA, CHROMagar, Paris, France) overnight at 35°C. Species identification was performed using the MALDI-TOF MS Biotyper (Bruker, Bremen, Germany). Susceptibility testing was performed using the EUCAST disc diffusion methodology (http://www.eucast.org). For cefoxitin-resistant isolates, the MRSA phenotype was confirmed genotypically with a PCR detecting the *mecA* or *mecC* gene with an in-house assay or Cepheid Xpert^®^ MRSA NxG assay (Sunnyvale, CA, USA).

For national surveillance, all first-time MRSA isolates from a patient were sent to the National Reference Laboratory for Antimicrobial Resistance at Statens Serum Institute, where among others a PCR detecting the Panton–Valentine leucocidin (PVL) was performed.

### Data collection

Study data were collected and managed using Research Electronic Data Capture (REDCap) hosted at the Capital Region of Denmark.^13,14^ From the laboratory information system (LIS), we retrieved personal information (identification number, age, sex, address) and sample set data (sample date, anatomical location, clinical or screening sample). From the clinical module of the LIS system and the electronic patient records, we retrieved household data (household size, number of MRSA-positive household members) and, if available, clinical information on treatments, chronic skin conditions (such as eczema, fistulas) or foreign bodies (such as insulin pumps, tympanostomy tubes). From the reference laboratory, we gathered data regarding the PVL gene.

### Outcome and time-to-event analyses

The primary outcome was becoming MRSA free. In this study, it was defined as the first negative screening sample set collected >180 days after the *first* positive sample (i.e. index sample, which could be a clinical sample or a screening sample) or after the *first* decolonization treatment. Thus, in the time-to-event analyses, time 0 was set to 180 days after the index sample for the no-treatment groups. In the treatment groups, it was set to 180 days after initiation of the first decolonization treatment.

Although the Danish MRSA Guidelines since 2012 recommend control samples 1 and 6 months after decolonization treatment, the definition of becoming MRSA free is having an MRSA-negative sample set at least 6 months after treatment, and children following the wait-and-see approach half-yearly or yearly will be declared MRSA free based on one sample set. Furthermore, two screening sets were not recommended before 2012.

According to the Danish MRSA Guidelines, a person can be declared MRSA free even if there are MRSA-positive household members. This was therefore not considered, and data on household members showed only how many were MRSA positive—not if they were still positive when the child was declared MRSA free.

**Figure 1.**
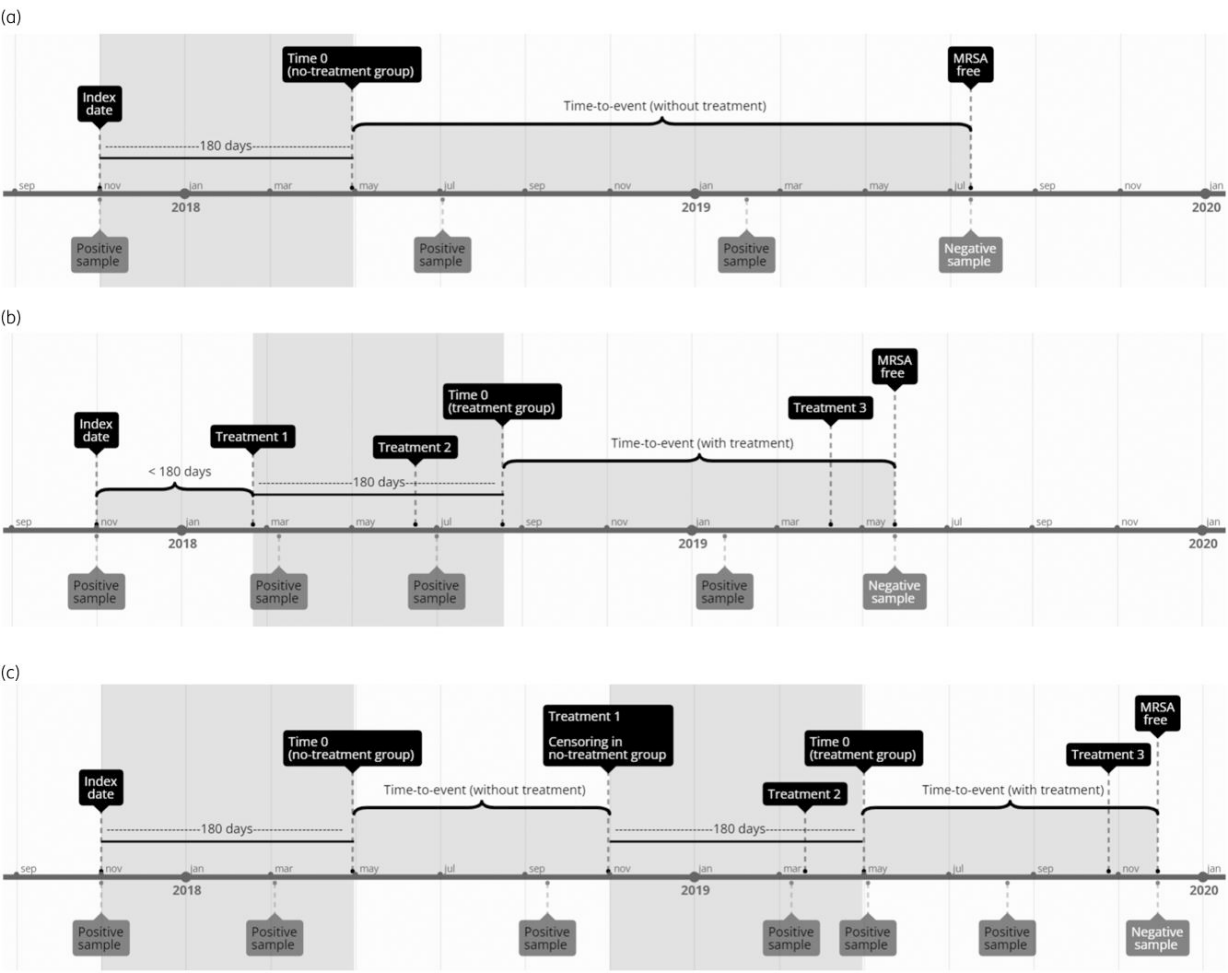
Simplified timeline examples. (a) Timeline where no decolonization treatment was given. The first 180 days after the index sample (grey shaded area) do not count in the time-to-event analyses, as a person cannot be declared MRSA free before 6 months. The child was declared MRSA free by the first negative sample set collected after this time 0. No censoring occurs in this example. (b) Timeline where decolonization treatment was given *less than* 180 days after the first positive sample. Time 0 is 180 days after the *first* decolonization treatment. No censoring occurs in this example. (c) Timeline where decolonization treatment was given *later than* 180 days after the first positive sample. The child was included in a no-treatment group 180 days after the index sample. Censoring followed when treatment was initiated, and the child was then included in a treatment group with time 0 at 180 days after the first treatment.

If more than 180 days passed between the first positive sample and the first decolonization treatment, the child was included first in a no-treatment group and subsequently in a treatment group (Figure 1c) (i.e. the treatment variable was handled as a time-dependent variable).

Participants were followed until becoming MRSA free, the end of follow-up or until being censored, whichever came first (Figure 1). Follow-up ended if the child reached an age of 2.5 or 6.5 years during the study period, for the two age groups, respectively. Censoring was done if a child was lost to follow-up, meaning no samples were collected for >24 months. We defined the loss to follow-up date as the date of the last sample set before the 24 month interval as proposed by Lesko *et al*.^15^ Censoring was also done at the time of treatment initiation if treatment was initiated after a child turned 2 or 6 years old, since there could not be >180 days follow-up. In the no-treatment groups, participants were censored if decolonization treatment was initiated and then included in the treatment group (Figure 1c). In the treatment groups, children were censored 1 year after the last decolonization treatment, as events later than this were unlikely to be a result of treatment.

### Exposure, variables, bias and confounders

Decolonization treatment was the primary exposure of interest. We defined it as ≥5 days of topical treatment with both chlorhexidine body washes and mupirocin nasal ointment, with or without the addition of systemic antibiotics. Treatments that did not follow a positive sample were not registered. Systemic treatment without concurrent topical decolonization treatment was not counted as decolonization treatment.

Colonized anatomical locations were determined based on samples collected before time 0 in the time-to-event analyses. The variable was grouped into nose positive (and not throat), throat positive (and not nose), both nose and throat positive, and ‘only perineum positive or nose and throat not tested’. The rationale for this grouping was that sample collection from nose and throat is the national recommendation, while perineal sampling is optional.

The fact that children 2–5 years old were routinely not treated if they had MRSA-positive <2-year-old household contacts could introduce bias, as they would be expected to carry MRSA for longer. Data were therefore adjusted for other MRSA-positive children <2 years old in the household. Other potentially confounding variables were sex, colonized anatomical locations, PVL gene, household size, number of MRSA-positive household members, whether the first positive sample was a clinical sample or a screening sample, chronic skin conditions, foreign bodies and treatment with relevant systemic antibiotics before time 0 in the time-to-event analyses. All confounders were included as baseline variables. Missing data were included in ‘unknown’ categories in the analyses, except for the variables chronic skin conditions and foreign bodies, where missing, were included in the ‘no’ category (Table 1).

**Table 1.** Epidemiological and clinical characteristics of study group

Age at first positive sample	<2 years old, *n* = 226		2–5 years old, *n* = 122	
Treatment group	No treatment, *n* = 170	Treatment, *n* = 56	No treatment, *n* = 32	Treatment, *n* = 90
Demographic data				
Age at inclusion (years), median (IQR)	0.12 (0.03–0.53)	0.13 (0.09–0.45)	3.84 (2.87–5.07)	3.85 (2.90–5.00)
Male sex, *n* (%)	102 (60)	27 (48)	16 (50)	42 (47)
Household size (persons), *n* (%)				
2–3	64 (38)	14 (25)	4 (13)	16 (18)
4–5	82 (48)	32 (57)	21 (66)	58 (64)
≥ 6	21 (12)	8 (14)	7 (22)	16 (18)
Unknown	3 (2)	2 (4)	0 (0)	0 (0)
Risk factors for prolonged carriage				
No. of other MRSA-positive household members, *n* (%)				
1–3	124 (73)	43 (77)	18 (56)	54 (60)
≥4	45 (26)	11 (20)	14 (44)	36 (40)
Unknown	1 (1)	2 (4)	0 (0)	0 (0)
Other MRSA-positive household members <2 years old, *n* (%)	34 (20)	15 (28)	20 (63)	20 (22)
Chronic skin disorders,^a^ *n* (%)	14 (8)	4 (7)	4 (13)	11 (12)
Foreign bodies,^a^ *n* (%)	4 (2)	1 (2)	0 (0)	5 (6)
Index sample was a clinical sample, *n* (%)	51 (30)	9 (16)	7 (22)	36 (40)
PVL gene detected, *n* (% of *n* tested)	29 (18)	7 (13)	10 (31)	27 (32)
Colonized location before time 0 in time-to-event analyses, *n* (%)				
Nose (and not throat)	36 (21)	10 (18)	10 (31)	22 (24)
Throat (and not nose)	23 (14)	4 (7)	9 (28)	14 (16)
Both nose and throat	103 (61)	39 (70)	11 (34)	49 (54)
Only perineum positive or nose or throat not tested	8 (5)	3 (5)	2 (6)	5 (6)
Treatment data				
No. of decolonization treatments, median (IQR)	—	1 (1–1)	—	2 (1–3)
Systemic decolonization treatment, *n* (% of all *n* treated)	—	31 (55)	—	31 (34)
Days from index sample to treatment, median (IQR)	—	55 (43–73)	—	18 (8–40)
Received systemic antibiotic treatment for an infection before time 0 in time-to-event analyses, *n* (%)	12 (7)	4 (7)	3 (9)	8 (9)

^a^We could not distinguish between negative and missing observations because they were only registered if present.

### Statistical analyses

In total, four subgroups of children were analysed and compared: age groups <2 years or ≥2 and <6 years old at the time of the first positive sample as well as treatment and no-treatment groups.

The probability of becoming MRSA free between the different groups was compared with Kaplan–Meier plots and Cox proportional hazards regression. Univariable Cox regression analyses were performed comparing the effect of the age group and of decolonization treatment. The multivariable regression analyses included all the mentioned potentially confounding variables. A subgroup analysis was performed comparing the probability of becoming MRSA free without treatment for children <3 months old versus children ≥3 months and <2 years old. The purpose of this subgroup analysis was to investigate specifically newborns, as the majority of these were presumed to be part of neonatal outbreaks. The proportional hazards assumption for each Cox regression analysis was evaluated with the Schoenfeld residuals method with a cut-off *P* value of <0.05.

Statistical analyses were performed using R (version 4.2.0) with RStudio (version 2022.07.01). The packages *tidyverse*, *data.table* and *lubridate* were used for data handling. The packages *survival* and *survminer* were used for the time-to-event analyses.^16,17^

The work was reported in accordance with the Strengthening the Reporting of Observational Studies in Epidemiology (STROBE) guidelines.^18^

### Ethics

The project was approved by the Capital Region’s Center for Regional Development (R-21048707) and was registered in the Region’s research inventory (P-2021-561).

## Results

**Figure 2.**
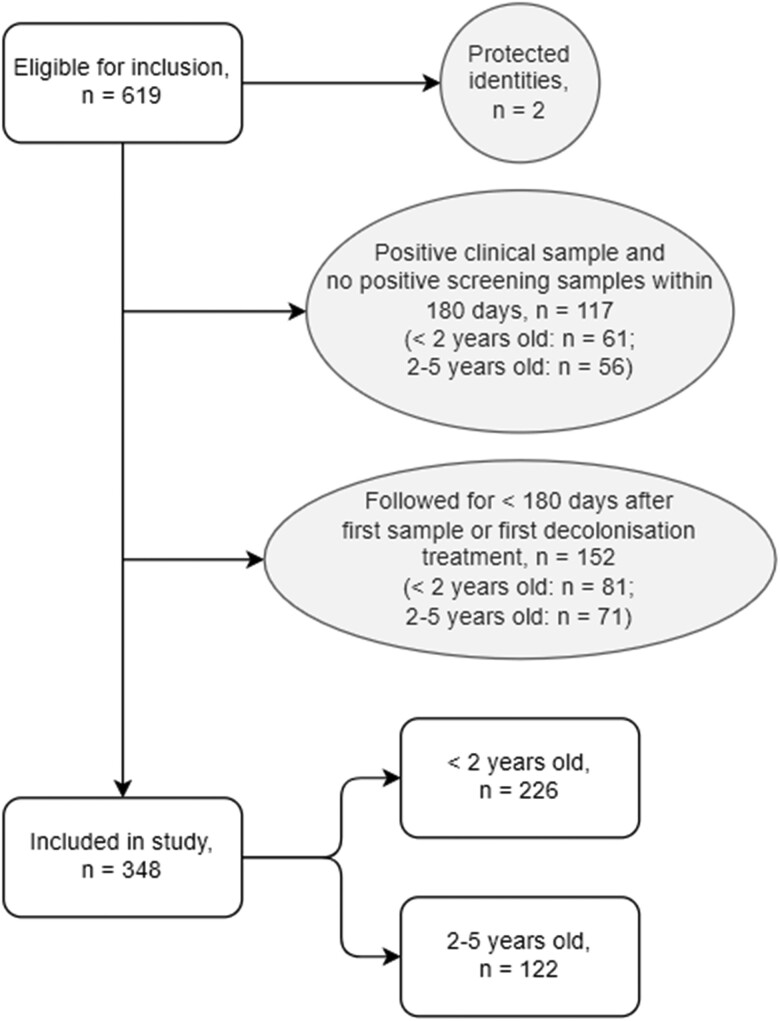
Flow diagram of eligible, included and excluded patients. Excluded patients are shown in oval boxes.

Of 619 children fulfilling the inclusion criteria, 348 were included in the study (Figure 2), 226/348 (65%) had an index age of <2 years and 122/348 (35%) had an index age of 2–5 years. Ten children were included in both treatment and no-treatment groups (Figure 1c), thus the total number in the time-to-event analyses was 358.

The age distribution of the whole cohort was skewed to the left, with 82/348 (24%) having their first positive sample within their first month of life, and 168/348 (48%) within their first 6 months.

Epidemiological and clinical characteristics of the 348 included patients are shown in Table 1. Of the <2-year-olds, 56/226 (25%) received decolonization treatment, and 28 of these children (50%) belonged to neonatal outbreaks. Of the 2–5-year-olds, 90/122 (74%) received treatment. While 31/56 (55%) of the <2-year-olds receiving decolonization treatment were treated with systemic antibiotics, this was only the case for 31/90 (34%) of the 2–5-year-olds. Of 73 systemic decolonization treatments (some children received >1 treatment), clindamycin was prescribed for 90%; the rest received clarithromycin, rifampicin combined with fusidic acid or trimethoprim/sulfamethoxazole.

Larger household sizes of 4–5 persons were more common among the 2–5-year-olds (64%–66%) than among the <2-year-olds (48%–57%), as well as household sizes of ≥6 persons (18%–22% versus 12%–14%). A larger proportion of the 2–5-year-olds had ≥ 4 MRSA-positive household contacts (40%–44% versus 20%–26%). The 2–5-year-olds in the no-treatment group had a larger proportion of <2-year-old MRSA-positive household contacts (63%) than all other groups, in accordance with recommendations to not treat household members of MRSA-positive children <2 years old. The PVL gene was detected more frequently among the 2–5-year-olds (31%–32%) than among the <2-year-olds (13%–18%).

In total, 188/358 (53%) children were declared MRSA free in the time-to-event analyses. Censoring was due to loss to follow-up (43/358; 12%), turning 2.5 or 6.5 years old or treatment initiated after age 2 or 6 years old (50/358; 14%), 12 months follow-up after the last decolonization treatment (62/358; 17%) or because decolonization treatment was initiated (15/358 (4%) [Figure 1c, Table S1 (available as Supplementary data at *JAC* Online)].

Of the 188 children who were declared MRSA free, 107 (57%) had at least two consecutive negative sample sets when being declared MRSA free. Only 58/188 (31%) were declared MRSA free based on a single sample and then not sampled again. However, 31/188 (16%) had positive samples after being declared MRSA free.

**Figure 3.**
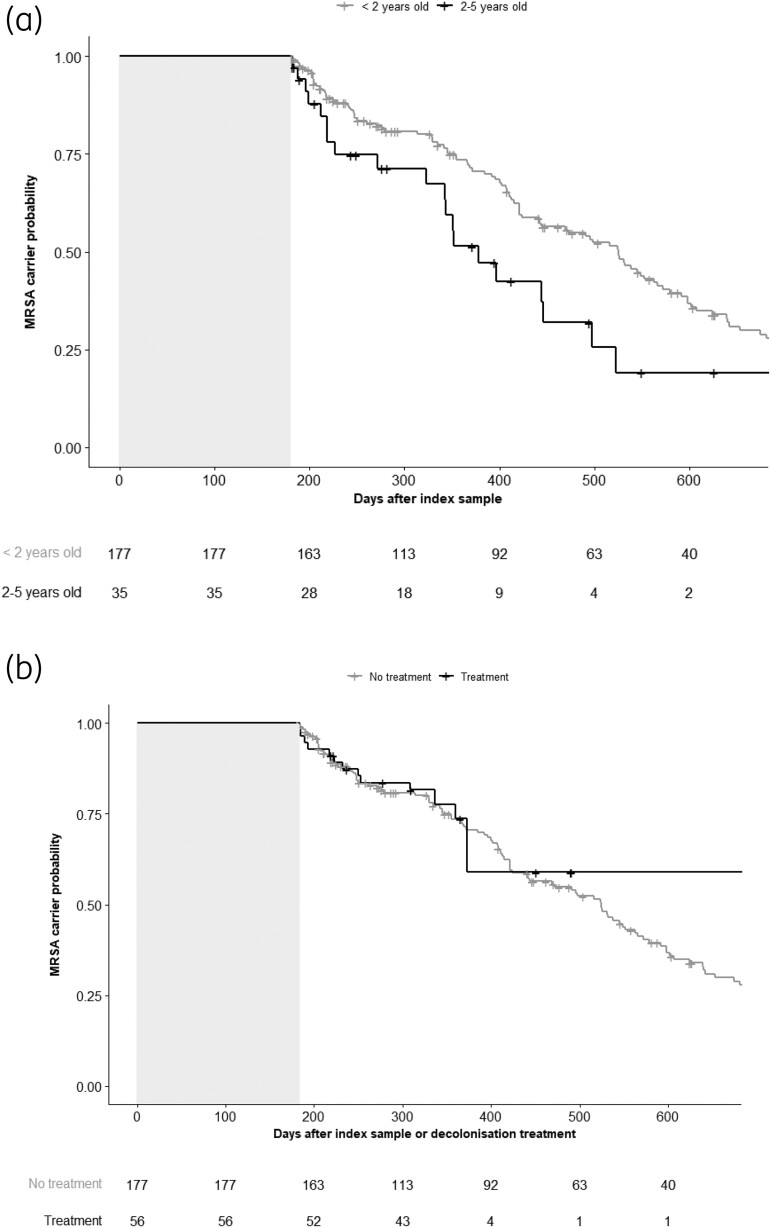

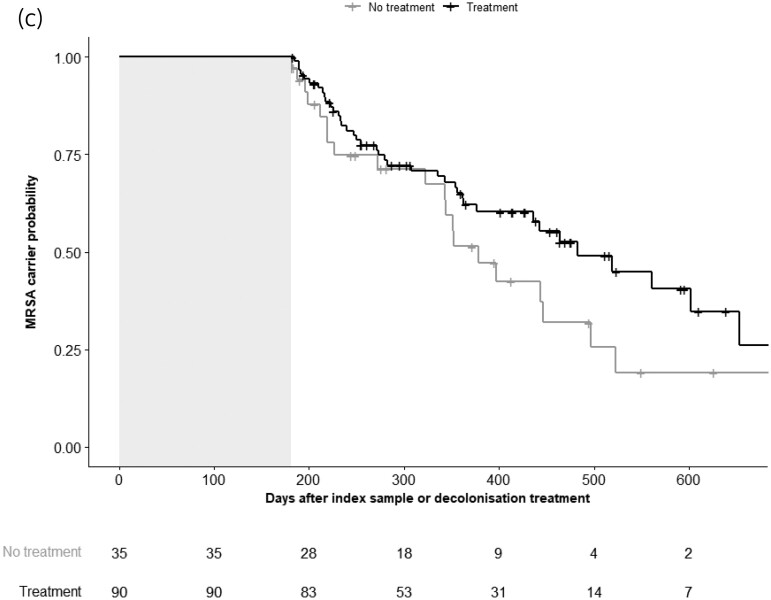
Probability of carrying MRSA illustrated with Kaplan–Meier plots. Time 0 is 180 days after the index sample or decolonization treatment (illustrated by grey shaded boxes). Numbers below the plots (risk tables) show the number of children still carrying MRSA at those timepoints. (a) Probability of becoming MRSA free without treatment depending on age group. (b) Probability of becoming MRSA free for <2-year-olds depending on decolonization treatment. (c) Probability of becoming MRSA free for 2–5-year-olds depending on decolonization treatment.

The median duration of MRSA carriage for <2-year-olds in the no-treatment group was 525 days; for 2–5-year-olds it was 378 days (Figure 3a). The median carriage duration was not estimated for <2-year-olds in the treatment group because this curve did not cross below a carrier probability of 50% (Figure 3b), while it was 483 days for the 2–5-year-olds (Figure 3c).

The univariable Cox regression analyses showed no larger effect of decolonization treatment versus no treatment in the <2-year-olds, with an HR of 0.97 (95% CI 0.56–1.69), or in 2–5-year-olds, with an HR of 0.62 (95% CI 0.36–1.06) (Table 2). The multivariable regression models showed similar results (Table 2).

**Table 2.** Results from Cox regression analyses, comparing HRs for becoming MRSA free in different groups

Groups	Univariable Cox regression, HR (95% CI)	*P* value	Multivariable Cox regression,^a^ HR (95% CI)	*P* value
<2-year-olds, treatment versus no treatment (ref.)	0.97 (0.56–1.69)	0.91	0.92 (0.52–1.65)	0.78
2–5-year-olds, treatment versus no treatment (ref.)	0.62 (0.36–1.06)	0.078	0.54 (0.26–1.12)	0.098
No treatment, 2–5-year-olds versus <2-year-olds (ref.)	1.86 (1.15–2.99)	0.011	1.81 (0.98–3.37)	0.059

^a^The multivariable regression analyses were adjusted for sex, colonized anatomical locations, detection of the PVL gene, household size, the number of MRSA-positive individuals in the household, MRSA-positive children <2 years old in the household, whether the first sample was a screening or clinical sample, chronic skin conditions, and whether systemic antibiotics were given before inclusion in the time-to-event analyses.

Comparing children of each age group who did not receive decolonization treatment, 2–5-year-olds became MRSA free faster than <2-year-olds, with an HR of 1.86 in the univariable analysis (95% CI 1.15–2.99). We also found this age group effect in the multivariable regression analysis, but it was not statistically significant (HR 1.81, 95% CI 0.98–3.37) (Table 2).

A subgroup analysis was undertaken for children who were <3 months old at the time of their first positive sample. Their median carriage duration was 522 days (Figure S1). For children who were ≥3 months and <2 years old at the first positive sample, the median carriage duration was 400 days. A univariable Cox regression analysis showed that children <3 months old at the first positive sample became MRSA free significantly more slowly than children ≥3 months and <2 years old (HR 0.58, 95% CI 0.39–0.87).

## Discussion

### Main results

Our results indicate that MRSA decolonization treatment is no more effective than no treatment in children <2 and 2–5 years old. Among children who did not receive decolonization treatment, 2–5-year-olds tended to become MRSA free faster than <2-year-olds. Thus, there was no evidence that <2-year-olds spontaneously clear MRSA carriage faster than 2–5-year-olds; in fact, rather the opposite.

According to Danish national guidelines, there was an expected larger proportion of children in the group of 2–5-year-olds receiving decolonization treatment. Many 2–5-year-olds who were not treated came from households with MRSA-positive siblings <2 years old (63%). Other reasons for not receiving treatment could be skin disorders or, less commonly, foreign bodies contraindicating treatment. The lower number of PVL-positive samples among the <2-year-olds can likely be attributed to several outbreaks in NICUs with PVL-negative clones, while the 2–5 years old often had community-acquired MRSA with a higher prevalence of PVL-positive clones.

### Comparison with other studies

Other studies have investigated the duration of MRSA colonization and effect of MRSA decolonization treatments with a variety of treatment and testing regimens and definitions of becoming MRSA free.^19,20^

A systematic review including 16 studies reported a median time to spontaneous MRSA clearance of 88 weeks (616 days); limitations were heterogeneous study characteristics and study populations.^19^ One study reported that in infants carrying MRSA at 6 days of age and receiving no decolonization treatment, 36.4% carried MRSA after 3 months, 22.6% after 6 months and 14.3% after 12 months.^21^

A Swedish study investigated decolonization treatment in children in a setting comparable to our setting but studied age groups of 0–6 and 7–17 years.^2^ Their results on the natural course of MRSA colonization align with ours, with 62% of the untreated children 0–17 years old carrying MRSA after 1 year and 28% after 2 years, with a median duration of 14.9 months. Successful decolonization was achieved for 36% of children receiving topical treatment only and 65% with the addition of systemic treatment.^2^

Few studies investigating decolonization treatment have used time-to-event analyses. MRSA colonization decreases spontaneously with time,^2,19^ consequently, it may be essential to include a time factor when evaluating the effect of decolonization treatment. A study from Sweden used this approach—among patients with a median age of 28 years (range 0–93 years), those receiving topical decolonization treatment had a significantly shorter duration of colonization and patients receiving systemic treatment even shorter. Also, children 0–17 years old were colonized for significantly longer than older patients, suggesting that treatment was less effective in this age group.^4^

Recent Danish studies on the effectiveness of the MRSA decolonization treatment regimen have not included children <2 years old as treatment of these children has not been recommended since 2012. These studies both found cumulative decolonization rates of approximately 62%–70% after up to four rounds of treatment.^3,22^

### Strengths and limitations

Strengths of this study include the large study size, the use of time-to-event-analyses and Cox regression models to adjust for potential confounders, and good data for decolonization treatments. The Danish personal identification number enabled us to easily combine data from different sources.

The study also has some important limitations. A clear limitation is that we required only one negative sample set to be declared MRSA free. Although the Danish MRSA Guidelines define being MRSA free as one negative sample set at least 6 months after decolonization treatment, it has been recommended since 2012 to collect two sample sets after completing treatment.

Accepting only one negative sample set at least 180 days following treatment was chosen as a pragmatic approach, as for children not receiving treatment, only one sample set is collected. We still believe this study provides new knowledge about the natural course of MRSA colonization and decolonization treatment in the two age groups since the same definition of being MRSA free was used for all groups. Requiring two negative sample sets in the analyses could also introduce falsely long carriage durations in case of irregular or long sampling intervals, or if a child was later recolonized.

Relying on only one negative screening set, the risk of a false-negative result could potentially influence our findings. We did, however, find that 57% had two consecutive negative sample sets when being declared MRSA free, supporting that the study results can be used to inform current clinical practice.

For methodological reasons, we had to disregard if children had positive samples after being declared MRSA free. Whether these were due to false-negative samples or recolonization from household members is not possible to determine from our data, but it would be the same for all groups. It has been demonstrated that the household plays a key role in MRSA transmission.^23^

There was a risk of unmeasured confounding, e.g. we did not have data on socioeconomic status, residency status and non-registered health conditions, and could therefore not adjust for these. The fact that some 2–5-year-olds could have received decolonization treatment because of MRSA-positive household members before being tested could also introduce bias as they might have been less likely to clear MRSA having not done so in the first treatment attempt (these treatment data for household members were not available). Since the first decolonization treatments were typically initiated by GPs, it may have been insufficiently registered in available clinical records. Data regarding skin disorders, foreign bodies and systemic treatment against infections were likely incomplete.

Recommended test intervals changed during the 14 year study period and were not standardized for all groups. For children with a wait-and-see-approach, samples were usually collected every 6–12 months, whereas after treatment, samples were usually collected after 1 and 6 months. A longer test interval could result in an estimated longer carriage duration. However, we did not find longer carriage durations for those children who followed the wait-and-see approach compared with those who received decolonization treatment.

Overall, the treatment groups were heterogeneous, and numbers of participants were low in the groups of <2-year-olds receiving treatment and 2–5-year-olds not receiving treatment. The inclusion bias that some 2–5-year-olds were not treated because of an MRSA-positive <2-year-old household member would decrease their risk of becoming MRSA free. The true probability of becoming free may therefore be even larger than the data show.

Giving decolonization treatment to <2-year-olds was standard practice before 2012, and overall, 50% of them were part of NICU outbreaks. Distinctive conditions pertaining to their underlying conditions, hospital admission, antibiotic treatments and testing patterns challenge the comparison between these children and other otherwise healthy children of the same age carrying MRSA. Fifty-five percent of <2-year-olds in the treatment group received systemic decolonization treatment, presumably because this was standard practice for neonatal unit outbreaks. The subgroup analysis of children <3 months old, of whom many were also part of neonatal outbreaks, shows that these children had a different natural course, being slower to clear MRSA spontaneously than children ≥3 months and <2 years old. The left skewed age distribution suggests that this had a significant impact on the age group.

### Generalizability

The Danish guidelines are specific with regard to children. The success of MRSA decolonization depends on decolonization treatment of the household, which influences the risk of recolonization. Because of this, the results are not easily transferable to a setting without a search-and-destroy strategy.

## Conclusions

MRSA decolonization treatment did not have a larger effect than no treatment in <2-year-olds or 2–5-year-olds. Without treatment, 2–5-year-olds tended to become MRSA free faster than <2-year-olds. These results support a wait-and-see approach for <2-year-olds and warrant considering the same approach for all children <6 years old. Further research is needed in older children to determine when they start to resemble the adult population with regard to the natural history of MRSA colonization and the effect of decolonization treatment.

## Supplementary Material

dkae036_Supplementary_Data

## Supplementary data

Figure S1 and Table S1 are available as Supplementary data at *JAC* Online.
