# Supplementary material for: Carriage of methicillin-resistant Staphylococcus aureus in children <6 years old: a retrospective follow-up study of the natural course and effectiveness of decolonization treatment
Source: J Antimicrob Chemother. 2024 Feb 9;79(4):826–34. doi: 10.1093/jac/dkae036 (PMC10984942; doi:10.1093/jac/dkae036)
Supplement: dkae036_Supplementary_Data [file dkae036_supplementary_data.docx]

**Table S1: Outcomes in the time-to-event analyses.**

| Age at first positive sample | <2 years old, n = 233 | | 2-5 years old, n = 125 | | All*,  N = 358 |
| --- | --- | --- | --- | --- | --- |
| Treatment group | No treatment,  n = 177 | Treatment,  n = 56 | No treatment,  n = 35 | Treatment,  n = 90 |  |
| MRSA free: event, n (%) | 112 (63) | 16 (29) | 21 (60) | 39 (43) | 188 (53) |
| Censored: Lost to follow-up, n (%) | 29 (16) | 1 (2) | 4 (11) | 9 (10) | 43 (12) |
| End of follow-up/censored: 2.5 or 6.5  years *or* treatment after 2 or 6 years,  n (%) | 26 (15) | 5 (9) | 5 (14) | 14 (16) | 50 (14) |
| Censored: Treatment started, n (%) | 10 (6) | - | 5 (14) | - | 15 (4) |
| Censored: End of follow-up one year  after treatment, n (%) | - | 34 (61) | - | 28 (31) | 62 (17) |
|  |  |  |  |  |  |
| * For 10 children, more than 180 days passed between the index sample and the initiation of decolonisation treatment. These children were included in both treatment and no-treatment groups, thus the total number of individuals in the time-to-event analyses was 358, although the number of unique study participants was 348. | | | | | |

**Figure S1: Probability of becoming MRSA free in the subgroup analysis, illustrated by Kaplan-Meier plots: Comparing children <3 months old to children ≥3 months and <2 years old.**

Time 0 is 180 days after the index sample or decolonisation treatment (illustrated by grey shaded box).

Numbers below the plot (risk table) show the number of children still carrying MRSA at those time points.

**
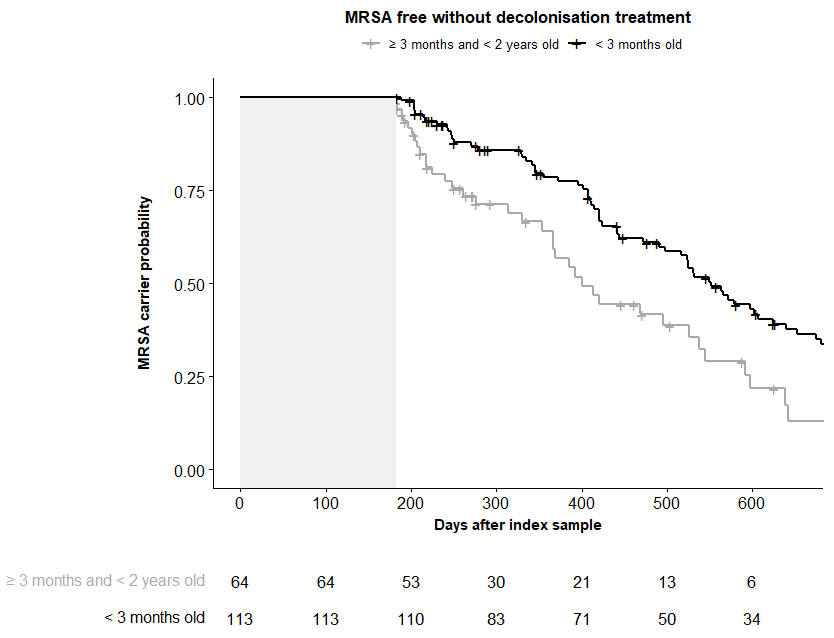
**
